# Alteration of Blood–Brain Barrier Integrity by Retroviral Infection

**DOI:** 10.1371/journal.ppat.1000205

**Published:** 2008-11-14

**Authors:** Philippe V. Afonso, Simona Ozden, Marie-Christine Cumont, Danielle Seilhean, Luis Cartier, Payam Rezaie, Sarah Mason, Sophie Lambert, Michel Huerre, Antoine Gessain, Pierre-Olivier Couraud, Claudine Pique, Pierre-Emmanuel Ceccaldi, Ignacio A. Romero

**Affiliations:** 1 Unité d'Epidémiologie et Physiopathologie des Virus Oncogènes, CNRS URA3015, Institut Pasteur, Paris, France; 2 Unité de Recherche et d'Expertise Histotechnologie et Pathologie, Institut Pasteur, Paris, France; 3 Laboratoire de Neuropathologie, Hôpital de la Salpêtrière, Paris, France; 4 Facultad de Medicina, Universidad de Chile, Santiago de Chile, Chile; 5 Department of Life Sciences, The Open University, Milton Keynes, United Kingdom; 6 Département de Biologie Cellulaire, CNRS 8104/INSERM 567/Paris V, Institut Cochin, Paris, France; University of Geneva, Switzerland

## Abstract

The blood–brain barrier (BBB), which forms the interface between the blood and the cerebral parenchyma, has been shown to be disrupted during retroviral-associated neuromyelopathies. Human T Lymphotropic Virus (HTLV-1) Associated Myelopathy/Tropical Spastic Paraparesis (HAM/TSP) is a slowly progressive neurodegenerative disease associated with BBB breakdown. The BBB is composed of three cell types: endothelial cells, pericytes and astrocytes. Although astrocytes have been shown to be infected by HTLV-1, until now, little was known about the susceptibility of BBB endothelial cells to HTLV-1 infection and the impact of such an infection on BBB function. We first demonstrated that human cerebral endothelial cells express the receptors for HTLV-1 (GLUT-1, Neuropilin-1 and heparan sulfate proteoglycans), both *in vitro*, in a human cerebral endothelial cell line, and *ex vivo*, on spinal cord autopsy sections from HAM/TSP and non-infected control cases. *In situ* hybridization revealed HTLV-1 transcripts associated with the vasculature in HAM/TSP. We were able to confirm that the endothelial cells could be productively infected *in vitro* by HTLV-1 and that blocking of either HSPGs, Neuropilin 1 or Glut1 inhibits this process. The expression of the tight-junction proteins within the HTLV-1 infected endothelial cells was altered. These cells were no longer able to form a functional barrier, since BBB permeability and lymphocyte passage through the monolayer of endothelial cells were increased. This work constitutes the first report of susceptibility of human cerebral endothelial cells to HTLV-1 infection, with implications for HTLV-1 passage through the BBB and subsequent deregulation of the central nervous system homeostasis. We propose that the susceptibility of cerebral endothelial cells to retroviral infection and subsequent BBB dysfunction is an important aspect of HAM/TSP pathogenesis and should be considered in the design of future therapeutics strategies.

## Author Summary

The blood–brain barrier (BBB) forms the interface between the blood and the central nervous system (CNS). BBB disruption is considered to be a key event in the pathogenesis of retroviral-associated neurological diseases. The present paper deals with the susceptibility of the endothelial cells (i.e., one of the main cellular components of BBB) to retroviral infection, and with the impact of infection in BBB function. This study focuses on the Human T-Lymphotropic Virus (HTLV-1), which infects 20 million people worldwide, and is the etiological agent of a neurodegenerative disease called HTLV-1 Associated Myelopathy/Tropical Spastic Paraparesis (HAM/TSP). We first demonstrated that the cerebral endothelial cells express the receptors for the retrovirus *in vitro*, and on spinal cord autopsy sections from non-infected and HAM/TSP patients. We found on these latter that vascular-like structures were infected and confirmed *in vitro* that the endothelial cells could be productively infected by HTLV-1. We demonstrated that such an infection impairs BBB properties *in vitro*, as well as tight junctions, that are cell adhesion structures. This study is the first to demonstrate the impact of HTLV-1 infection on human BBB integrity; such a susceptibility has to be considered in the design of future therapeutics strategies.

## Introduction

The Blood-Brain barrier (BBB) constitutes the interface between the blood and the central nervous system (CNS). It is composed of astrocytes, pericytes and brain microvascular endothelial cells. This latter cell type forms the major structural and functional element of the BBB, with endothelial cells sealed together with Tight Junctions (TJs). Under physiological conditions, the BBB maintains CNS homeostasis and selectively regulates intracellular and paracellular passage of ions, molecules and cells [1].

BBB integrity is compromised during retroviral infection; for example, BBB breakdown has been reported during Human Immunodeficiency Virus Type 1 (HIV) infection, especially during HIV-related encephalitis and HIV-associated dementia [2]. One to three percent of the 20 million people infected worldwide by the retrovirus HTLV-1 (for human T-lymphotropic virus type 1) develop HTLV-Associated Myelopathy/Tropical Spastic Paraparesis (HAM/TSP) [3]. This is a slowly progressive paraplegia of the lower extremities, involving demyelination and neuronal degeneration mainly in the thoracic spinal cord. BBB disruption has been attested in HAM/TSP patients by several lines of evidence, such as fibrinogen leakage and IgG deposits in CNS parenchyma [4] as well as lymphocyte passage through brain endothelium [4]–[7]. As previously shown, BBB disruption is associated with alterations in tight junctions between endothelial cells in the vasculature of a HAM/TSP patient [8].

The mechanisms of BBB disruption during retroviral-associated pathologies are not yet fully understood. Most studies focus on the effect of soluble molecules secreted by infected lymphocytes on BBB functions and intercellular TJ organization. In the case of HIV infection, the viral protein Tat has been shown to induce an inflammatory process in brain endothelial cells, or endothelial cell apoptosis [9], and to be able to disrupt the intercellular TJs [10]. In the context of HTLV-1 infection, we recently demonstrated that proinflammatory cytokines, such as IL-1α and TNFα, secreted by infected lymphocytes, are sufficient to disrupt TJs between human brain endothelial cells and induce permeability changes [8].

Alternative mechanisms could contribute to BBB dysfunction associated with HTLV-1 infection. Although neurological disease in mice infected with the PVC-211 Murine Leukemia Virus has been associated with infection of brain endothelial cells [11], the infection of brain endothelial cells by human retroviral agents and its role in BBB breakdown is still a matter for debate. In the case of HIV infection, a number of earlier studies reported infection of endothelial cells in adult brain tissue [12]–[14], based upon morphological appearance and vascular localization of cells found positive by immunocytochemistry, *in situ* hybridization or PCR-*in situ* hybridization for viral transcripts. Conflicting results were obtained *in vitro* from brain-derived endothelial cells (for review, see [15]).

In the case of HTLV-1, no evidence for infection of human brain endothelial cells has been reported so far, most likely due to the rarity of material from patients with HAM/TSP, and the low level of HTLV-1 expression in tissues. Although an increased adherence of T lymphocytes from HAM/TSP patients to human brain endothelial cells has been observed [16], the main data concern extra-neural endothelial cells: it has been demonstrated *in vitro* that human venous endothelial cells derived from umbilical cords are susceptible to HTLV-1 infection [17],[18], and that HTLV-1 proviral DNA could be detected in dermal endothelial cells *ex vivo* [19].

In this study, we investigated the susceptibility of human brain endothelial cells to HTLV-1 infection, and its possible consequences on BBB integrity, both *in vitro*, in a human brain endothelial cell line, and *ex vivo* on spinal cord autopsy sections from HAM/TSP patients. We found that human brain endothelial cells can be productively infected *in vitro* by HTLV-1, with consequent alterations in the BBB, evidenced by increased lymphocyte migration and passage of small molecules through endothelium. These data provide a basis for and transient BBB alterations that may be observed during BBB pathogenesis.

## Results

### Expression of HTLV-1 receptors within the spinal cord of uninfected or HAM/TSP patients

Three cellular components have been identified as forming part of the HTLV-1-entry complex: heparan sulfate proteoglycans (HSPGs) [20],[21], Neuropilin-1 [22], a co-receptor for VEGF165 and semaphorin 3a, and the glucose transporter Glut-1 [23].

The expression of HSPGs in BBB endothelial cells has previously been reported; *in vivo*, HSPGs are ubiquitously expressed at the cell surface or throughout the extracellular matrix of all mammalian tissues [24]; in particular, HSPGs have been previously detected in cerebral blood vessels [25].

In this study, we examined the expression of Glut-1 and Neuropilin-1 in the endothelial cells that form the BBB *in situ*. As HAM/TSP is characterized by lymphocyte infiltration and inflammation mainly within the thoracic spinal cord, we focused our investigation on this region.

**Figure 1:**
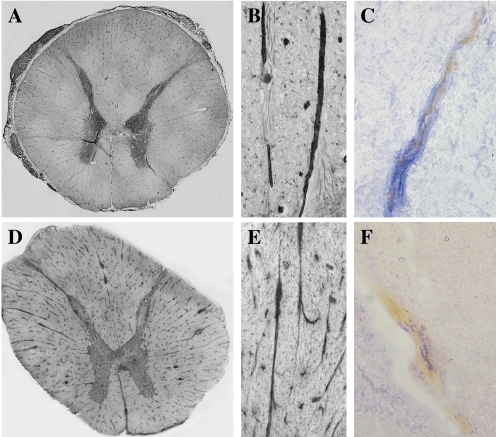
Expression of HTLV-1 receptors, Glut-1 and NP-1, in the thoracic spinal cord from an uninfected individual. (A,B) Glut-1 immunostaining by immunoperoxydase technique (iPO) (DAB substrate). (C) Dual staining for Glut-1 (iPO, DAB substrate, brown color) and factor VIII (Alcaline phosphatase (AP), FastBlue substrate, blue color). (D,E) NP-1 staining by iPO technique (DAB substrate). Thoracic spinal cord specimens were cut in a cryostat, fixed in methanol and processed for iPO as described in Materials and Methods. (F) Dual staining for NP-1 (iPO, DAB substrate, brown color) and factor VIII (Alcaline phosphatase, FastBlue substrate, blue color). Frozen tissue samples from the thoracic spinal cord were cut on a cryostat at 10 µm, fixed in methanol and processed for immunohistochemistry as described in Materials and Methods. Magnification: (A,D) 5×, (B–E) 50×, (C–F) 100×.

In tissue sections derived from the thoracic spinal cord of uninfected control cases, Glut-1 expression was diffuse and patchy on fibres throughout the grey matter, intense on fibers in the dorsal root and also detected in the meninges surrounding the cord (Fig. 1A). Glut-1 was expressed prominently on blood-vessels within both the white and the grey matter (Fig. 1A and B). The Neuropilin-1 (NP-1) was expressed diffusely in the posterior, lateral and anterior columns, and in the dorsolateral fasciculus/dorsal root (Fig. 1D). Cellular expression was particularly noted at the apex of the posterior column, and on motor neurons in the anterior column (data not shown). Significantly, NP-1 was highly expressed on blood vessels, and within the meninges in all segments of the thoracic spinal cord (Fig. 1D and E). Vascular endothelial cell expression of Glut-1 and NP-1 was confirmed by double immunolabeling with Factor VIII, a specific marker for endothelial cells (Fig. 1C and F).

**Figure 2:**
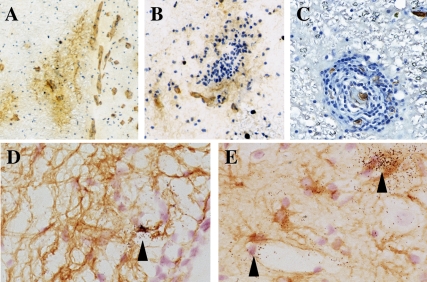
Detection of Glut-1 and HTLV-1 transcripts in the thoracic spinal cord from HAM/TSP patient. (A,B,C) Glut-1 staining by iPO (DAB substrate, brown) on thoracic spinal cord sections from a HAM/TSP patient. Nuclei were counterstained with Harri's haematoxilin solution (blue). (A,B) Cryostat section; (C) paraffin section. Magnification: (A) 30×; (B,C) 75×. (D,E) Detection of HTLV-1 transcripts (tax) in cryosections of the spinal cord by *in situ* hybridization. Arrow heads indicate positive cells and vascular structures. Astrocytes were detected by iPO (DAB substrate, brown) against GFAP. Magnification: 220×.

We then determined whether expression of these receptors by endothelial cells was conserved in HAM/TSP. In tissue sections of the thoracic spinal cord derived from HAM/TSP patients, Glut-1 immunoreactivity was detected in blood vessels, in the absence (Fig. 2A) or presence of cell infiltrates (Fig. 2B and C). Neuropilin-1 could also be detected in blood vessels in sections from HAM/TSP patients (data not shown).

Since microvascular endothelial cells that constitute the BBB express HTLV-1 receptors, we examined whether the infection of these cells by HTLV-1 could be detected *in situ*, by performing *in situ* hybridization for a viral mRNA (the messenger that encodes the viral transactivator Tax) on the spinal cord sections. Cellular infiltrates were positive for viral Tax mRNA (data not shown). However, we focused our analyses on spinal cord regions where the infiltrates were absent, to prevent the signal within the infected lymphocytes from masking the signal from resident cells within the CNS parenchyma. Since astrocytes are known to be targets of HTLV-1 infection [26],[27], the detection of a positive signal in several GFAP immunoreactive cells constituted a suitable positive control (Fig. 2D), as shown in previous studies [4]. We also encountered rare positive signals associated with vascular structures (Fig. 2E). This observation suggested the possibility of infection of cerebral endothelial cells by HTLV-1.

### Expression of HTLV-1 receptors by hCMEC/D3 cells

Although viral transcripts were found to be associated with blood vessels, this observation could not be taken as definitive evidence for the infection of endothelial cells forming the BBB specifically as other cell types such as pericytes are closely associated with endothelial cells. We therefore took advantage of a human-derived brain endothelial cell line, hCMEC/D3, that had been previously reported to retain many BBB characteristics [28]. We first investigated the expression of the 3 (co-)receptors for HTLV-1 entry by flow cytometry analysis.

**Figure 3:**
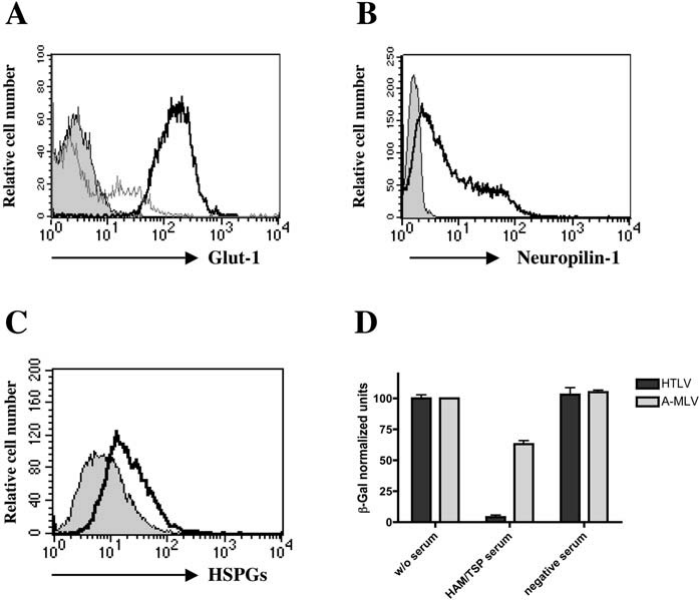
Expression of viral receptors at the cell surface of hCMEC/D3 cells and susceptibility to infection by HTLV-Env pseudotyped particles. (A) FACS analysis of Glut-1 expression on hCMEC/D3 cells. Signal for permeabilized cells appear as bold-lined curve; unpermeabilized as thin-lined curve; control cells (without Glut-1 antibody) appear as a grey-filled curve. (B) FACS analysis of Neuropilin-1 expression on hCMEC/D3 cells. Signal for unpermeabilized as bold-lined curve; control cells (without NP-1 antibody) appear as a grey-filled curve. (C) FACS analysis of Heparan sulfate-proteoglycans expression on hCMEC/D3 cells. Signal for unpermeabilized as bold-lined curve; control cells (without HSPGs antibody) appear as a grey-filled curve. (D) Infection assay on hCMEC/D3 cells using *Lac-Z-*carrying MLV particles pseudotyped with the HTLV (HTLV) or Amphotropic (MLV) envelope proteins. β-gal production was measured using chemiluminescence after subtracting the background of uninfected cells on total protein normalization. Infections were performed without serum, or in the presence of serum from a HAM/TSP patient (HAM/TSP) (1/10) or from a HTLV-uninfected individual (control) (1/10). Results represented as means±SD.

Glut-1 expression was highly detected in permeabilized hCMEC/D3 cells. In order to detect expression of this protein at the cell surface, immunostaining was performed on fixed but non-permeabilized cells. The expression of Glut-1 at the cell surface was detected in 31% of the hCMEC/D3 cells (Fig. 3A). Similarly, cell surface expression of NP-1 and HSPGs were detected in 82% and 59% of the hCMEC/D3 cells respectively (Fig. 3B and C).

### Infection of human cerebral endothelial cells hCMEC/D3 cells by HTLV-1

In order to determine if the expression of surface receptors on endothelial cells allows HTLV-1 entry, we investigated whether hCMEC/D3 cells could be infected by HTLV Env-pseudotyped LacZ vectors. β-Gal production was detected upon infection of hCMEC/D3 cells by A-MLV as well as by H-MLV pseudotypes. Addition of serum from an uninfected donor did not reduce infection by either pseudotype. In contrast, addition of serum from an HTLV-1-infected HAM/TSP patient abolished the infection by H-MLV, while high level of A-MLV pseudotype infection was still observed (Fig. 3D). These results indicate that hCMEC/D3 cells allow HTLV Env-mediated entry.

**Figure 4:**
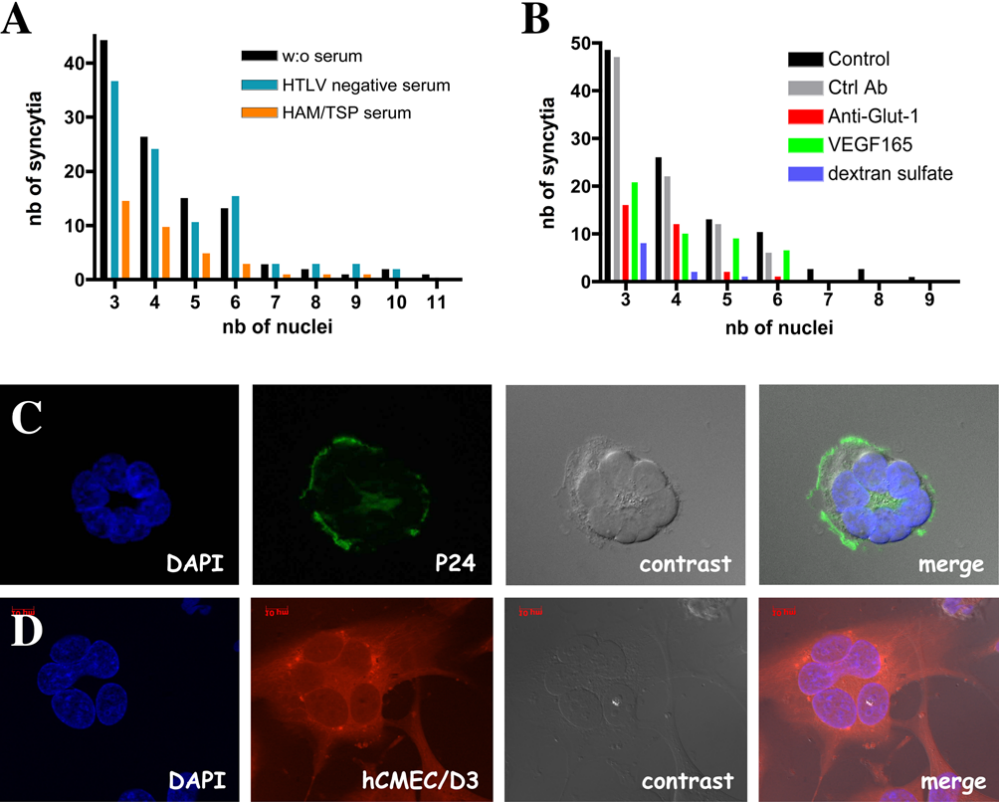
Syncytia formation between hCMEC/D3 cells and HTLV-1 infected MT-2 lymphocytes at 24 h post-contact. (A) Role of the viral proteins in the formation of the syncytia. Evaluation of the number and size (nb of nuclei/syncytium) of the syncytia obtained by coculture between hCMEC/D3 and MT-2 cells in the presence of serum from a HAM/TSP patient (1/7) (noted HAM/TSP) or from an uninfected individual (control, noted HTLV negative control or in the absence of any serum (w/o serum). (B) Role of the viral receptors in the formation of the syncytia. Evaluation of the number and size (nb of nuclei/syncytium) of the syncytia obtained by coculture between hCMEC/D3 and MT-2 cells in the presence of dextran sulfate in blue (that prevents the HTLV-1 Env/HSPG interaction), or in the presence of VEGF165 in green (that prevents the HTLV-1 Env/NRP-1 interaction), or in the presence of a polyclonal antibody against Glut-1 in red (that prevents the HTLV-1 Env/GLUT1 interaction). Results are representative of 3 independent experiments. (C) Detection of viral p24 protein (green) by immunofluorescence in a syncytium. Nuclei were stained with DAPI (blue). (D) Demonstration of endothelial origin of the syncytia by prior labeling of hCMEC/D3 cells with a red vital fluorescent marker (Cell-tracker, red). Nuclei were stained with DAPI (blue). Magnification (B–C): 350×.

The ability of a retrovirus such as HTLV-1 to enter a particular cell type is usually correlated with the ability of target cells to fuse and form syncytia with infected T lymphocytes. We therefore quantified the number of syncytia in hCMEC/D3 and lymphocyte cocultures. Whereas hCMEC/D3 did not fuse with C81-66 lymphocytes (HTLV-1-infected cells that do not express Env glycoproteins), numerous syncytia were observed in co-cultures with infected MT2 lymphocytes at 24 hours. The number of nuclei per syncytium ranged from 3 to 11 (Fig. 4A). Syncytia were found immunoreactive for viral p24 (Fig. 4C). In order to ascertain the endothelial origin of the syncytia, we prestained the hCMEC/D3 cells with a vital fluorescent molecule (CellTracker Red CMTPX). Syncytia were shown to be fluorescently labeled (Fig. 4D).

Addition of serum from an uninfected patient did not prevent the formation of syncytia. In contrast, a dramatic reduction in the number and the size of syncytia was observed when serum from an HAM/TSP patient was added to the medium (Fig. 4A), showing that the fusion is HTLV-mediated. Similarly, syncytia formation could also be inhibited by addition of VEGF165, a physiological ligand of NRP-1 that has been shown to inhibit the binding of the HTLV-1 Env proteins to target cells [22], of dextran sulfate, an inhibitor of HSPG-mediated HTLV-1 entry [20], or of an antibody directed against the glucose transporter Glut-1 (Fig. 4B). It is worth noting that no significant alterations in syncytia formation were observed following addition of an irrelevant isotype-matched antibody to the culture medium.

These data suggest that HTLV-1 entry into hCMEC/D3 cells is dependent on the interactions between viral envelope proteins and the three putative cellular receptors for HTLV-1 infection (heparan sulfate proteoglycans, neuropilin-1 and Glut-1).

### Human cerebral endothelial cells are productively infected by HTLV-1

As HTLV-1 can enter hCMEC/D3 cells, we then determined whether this event allows a productive infection. Endothelial cells were co-cultivated with irradiated lymphocytes. The irradiation dose was lethal for these cells, as confirmed by Trypan blue staining which indicated that 100% of the lymphocytes were dead by day 8 post-irradiation (data not shown).

**Figure 5:**
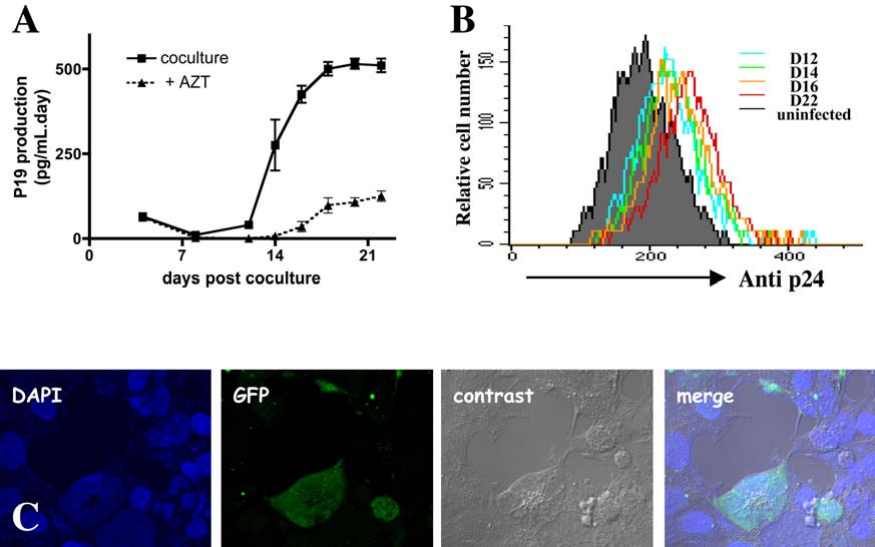
Productive infection of hCMEC/D3 cells by HTLV-1. (A) Kinetics of p19 viral protein secretion in the supernatant of hCMEC/D3 and irradiated HTLV-1 MT-2 lymphocyte cocultures. Cells were cultivated or not in presence of 25 µM AZT. Results are mean and standard deviation from triplicate experiments. (B) Kinetics of detection of infected hCMEC/D3 cells by irradiated MT-2 cells. Infection was assessed by FACS analysis of p24 viral protein at days 12, 14, 16 and 22 post-coculture. (C) Production of infectious viral particles by HTLV-1-infected endothelial cells using a reporter cell-line (293T-LTR-GFP). The supernatant of hCMEC/D3 infected cells was collected and ultracentrifuged and the resuspended pellet was applied on the reporter cell-line 293T-LTR-GFP. The expression of GFP was assessed 6 days later after cell fixation.

HTLV p19 was detected in the supernatants of co-cultures but decreased during the 10 first days: this correlated with lymphocyte and syncytia cell death (data not shown). At 10 days post coculture, no syncytium could be observed. From day 13 post-contact, p19 production to reach about 510 pg/day at day 22, indicating that human brain endothelial cells produced viral proteins (Fig. 5A). HTLV-1 productive infection could be prevented by addition of AZT, an inhibitor for the reverse transcriptase, to the culture medium. The percentage of positive endothelial cells for viral p24 increased in parallel to p19 levels detected in the supernatant reach maximal infectivity rates at day 22, with 18% of the endothelial cells positive for p24 (Fig. 5B). No cytopathic effect of HTLV-1 infection endothelial cells was observed.

At day 22 post co-culture (2 days after renewal of the medium), the supernatant was collected and ultracentrifuged. The pellet was resuspended and added to culture medium of reporter 293T-LTR-GFP cells. After 6 days of culture, fluorescent cells were visualized, demonstrating the presence of Tax protein within these reporter cells. Furthermore the fluorescent cells could form syncytia, and expressed the viral envelope protein (Fig. 5C). In addition, the number of the fluorescent cells was dramatically reduced (up to 37%) in the presence of AZT indicating that the detected fluorescence signal in 293T-LTR-GFP cells was specific to *de novo* infection of reporter cells. These results indicate that cerebral endothelial cells produced infectious viral particles.

### Impairment of BBB functions following infection by HTLV-1

Lastly, we assessed the impact of HTLV-1 infection of endothelial cells on BBB function, by evaluating the paracellular permeability of hCMEC/D3 cell monolayers and the transmigration of lymphocytes through the barrier in an infected and non-infected context.

**Figure 6:**
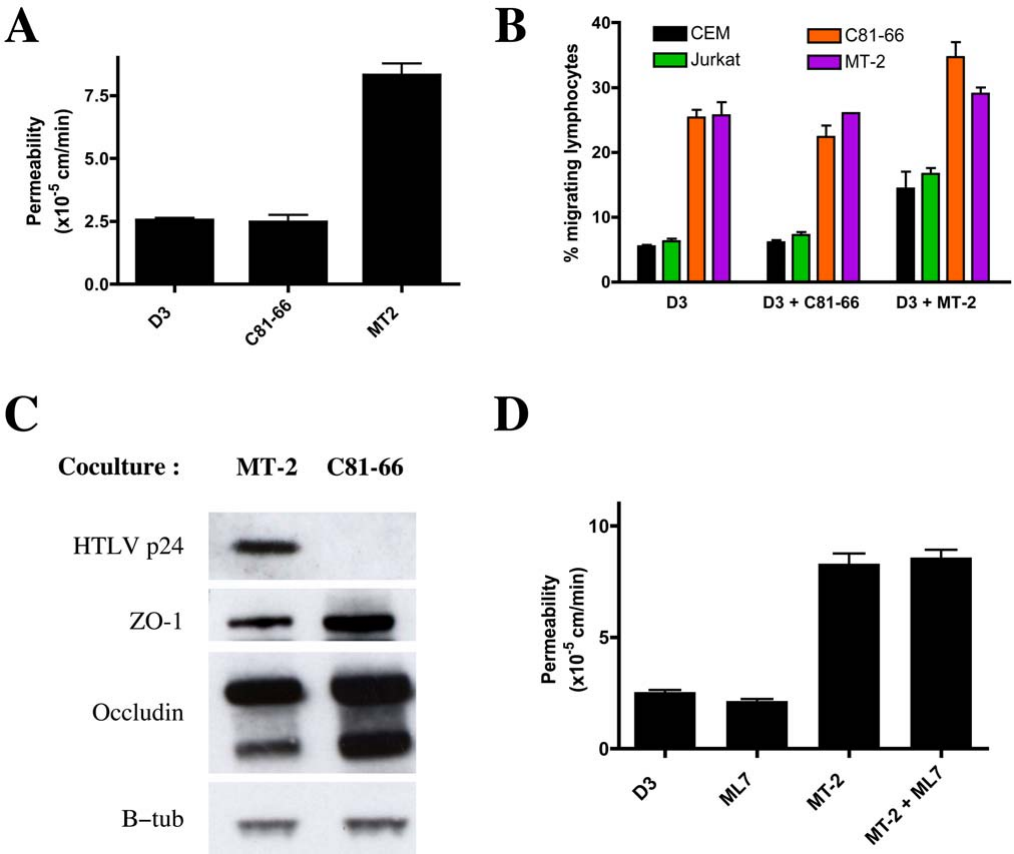
Alteration of BBB functions in the infected hCMEC/D3 cells. (A) Altered permeability of a monolayer of infected hCMEC/D3 cells. The endothelial cells were cocultured with irradiated MT-2 or C81-66 lymphocytes for 15 days. Then hCMEC/D3 cells were seeded on Transwell filters and permeability to FITC-dextran 70 kDa was assessed after differentiation of the monolayer. (B) Effect of endothelial cells infection on CEM lymphocyte migration. Infected endothelial cells were seeded onto filters. The migration was estimated at 24 hours of culture by fluorescence assay after labeling of lymphocytes (from the CEM, Jurkat, MT-2 of C81-66 cell-lines) with a fluorescent marker. The migration rate is expressed as ratio (%) of fluorescence intensity in the lower compartment versus total fluorescence. (C) Analysis of the expression level of ZO-1, Occludin, and viral p24 of a monolayer of hCMEC/D3 cells cocultured with irradiated MT-2 or C81-66 for 15 days. β-tubulin was used for normalization. (D) Inhibiting MLC phosphorylation has no effect on the permeability for FITC dextran 70 kDa across a monolayer of infected endothelial cells. hCMEC/D3 cells were seeded on filters. After differentiation, cultures were either left untreated of treated for 48 hours with ML-1, a specific inhibitor for MLCK activity. Permeability was then estimated as described in Materials and Methods.

hCMEC/D3 cells, incubated with irradiated C81-66 (HTLV-1-infected lymphocytes that are not productively infected) or with MT2 cells 15 days prior to the experiment were seeded on filters and allowed to reach confluence. The paracellular permeability of monolayers of hCMEC/D3 cells infected with HTLV-1 was much higher than the permeability of hCMEC/D3 cells previously cocultured with control C81-66 T-lymphocytes (Fig. 6A). Similarly, transmigration of uninfected T-lymphocytes (CEM and Jurkat) across monolayers of hCMEC/D3 cells infected with HTLV-1 was increased compared to that across a monolayer of uninfected hCMEC/D3 cells (Fig. 6B). In addition, the migration of HTLV-1-infected lymphocytic cell lines through hCMEC/D3 cells was increased compared to control lymphocytes as previously reported [8]. However, no differences in HTLV-1-infected lymphocyte transmigration across HTLV-1-infected or non-infected hCMEC/D3 cells were observed. These data indicate that HTLV-1-infection of endothelial cells alters classical BBB functions.

We then analyzed the expression levels of proteins that constitute the tight junctions by Western blot. As indicated in Fig. 6C, ZO-1 levels were dramatically reduced in HTLV-1-infected hCMEC/D3 cells whereas, in the case of occludin, the expression levels of the 55 kDa isoform, but not those of the 60 kDa isoform were decreased.

Since we have previously shown that the Myosin Light Chain Kinase (MLCK) is important in tight junction regulation of endothelial cells incubated with HTLV-1 infected lymphocytes, we determined whether the inhibition of MLCK activity could prevent long-term barrier impairment of HTLV-1 infected endothelial cells (Fig. 6D). Treatment of the endothelial cells for 24 h with the MLCK inhibitor ML7 failed to restore low paracellular permeability in HTLV-1 infected hCMEC/D3 cells. The molecular mechanisms of TJ disruption in endothelial cells induced by direct HTLV-1 infection appear to be different to those induced by HTLV-1-infected lymphocytes, as previously demonstrated [8].

## Discussion

The BBB constitutes an interface between the bloodstream and CNS parenchyma, and regulates the intracellular and paracellular passage of molecules and cells between the two compartments [1].

Disruption of the BBB in HAM/TSP is strongly suggested by observations of perivascular cuffing with lymphocyte and macrophage infiltrates [4],[5],[7], and confirmed by reports of fibrinogen and IgG deposits in the CNS parenchyma of HAM/TSP patients [4]. BBB breakdown is an important step in HAM/TSP pathogenesis, especially by facilitating migration of lymphocytes into the CNS. Infiltrated lymphocytes are believed to cause demyelination and axonal degeneration that are hallmarks of HTLV-1-associated neuropathology [29]. The mechanisms underlying BBB alteration during HAM/TSP are not yet well determined. In an *in vitro* model of the BBB, composed of human cerebral microvascular endothelial cells [28], we previously demonstrated the importance of proinflammatory cytokines secreted by infected lymphocytes in the early stages of BBB disruption [8],[30]. In the present study we investigated whether BBB endothelial cells were susceptible to HTLV-1 infection and the impact of such an infection on BBB integrity.

BBB dysfunction associated with retroviral infections has been previously described. In a murine model, the infection of brain endothelial cells has been reported both *in vitro* and *in vivo* in the case of PVC-211 murine leukemia virus (a neuropathogenic variant of the Friend MuLV), with a direct correlation between the replication efficiency of a virus in brain endothelial cells *in vitro* and its ability to cause neurological disease in vivo [11]. Moreover, in the case of infection by the Feline Immunodeficiency Virus, infection of brain endothelial cells has been proposed to represent one of the ways of viral entry into the CNS [31]. Meanwhile, infection of brain endothelial cells by human retroviral agents is still a matter of debate. In the case of HIV infection, previous studies have reported infection of endothelial cells in adult brain tissue [12]–[14], but these have been mainly based upon interpretation of the morphological appearance and vascular localization of cells found positive by immunocytochemistry, *in situ* hybridization or PCR-*in situ* hybridization, whereas conflicting results have been obtained *in vitro* from brain-derived endothelial cells (for review, see [15]). Up until now, no study has focused on the subject of human endothelial cell infection by HTLV-1.

Recently three membrane proteins have been described as components of the receptor for HTLV-1 entry: the glucose transporter Glut-1 [23], and a receptor for VEGF, Neuropilin-1 [22] and heparan sulfate-proteoglycans [21],[32]. Vascular expression of Glut-1 [33],[34] and Neuropilin-1 [35],[36] has been previously reported within the CNS, under normal and pathological conditions. We have shown that the HTLV-1 receptors are expressed on blood vessels of the adult human thoracic spinal cord, a region which is characterized by BBB impairment, lymphocyte infiltration and inflammation during HAM/TSP. Expression of these proteins was found in endothelial cells within the spinal cord of HAM/TSP patients, irrespective of the extent of lymphocyte infiltration. We could also detect expression of these receptors at the cell surface of a human cerebral endothelial cell line, hCMEC/D3.

We then looked for HTLV-1 infected endothelial cells by *in situ* hybridization using DNA probe directed against transactivator Tax transcripts on spinal cord sections of a HAM/TSP patient. Astrocytes are reportedly susceptible to HTLV-1 infection *in vitro* [26],[27]. Astrocytic infection with HTLV-1, also reported using this technique in CNS tissues (Ozden et al. 2002), served as a positive control in our samples. HTLV-1 transcripts were found only very rarely associated with the vasculature. The difficulty in detecting the virus within endothelial cells *in situ* may be in part due to the specific immune response developed against HTLV-1 in HAM/TSP patients [37]. HTLV-1 infected endothelial cells may be detected and lysed by cytotoxic lymphocytes, thereby constituting a new mechanism for transient BBB disruption, which remains to be explored further. Moreover, the tissue sections studied are from a patient with a rapid progressing HAM/TSP [4]. Additional studies in other HAM/TSP patients, with slower disease progression, might facilitate the detection of HTLV-1-infected endothelial cells.

As the *ex vivo* detection was difficult to ascertain, we studied the susceptibility of an *in vitro* model, hCMEC/D3, to HTLV-1 infection. We confirmed that the expression of the receptors at the cell surface of hCMC/D3 cells allowed viral entry. In fact, it has been demonstrated that cholesterol, a central component of lipid rafts, was necessary for HTLV-1 infection, especially on post-binding entry steps [38]. Thus we examined whether the spatial organization of viral receptors on the membrane of cerebral endothelial cells would enable infection with HTLV. Using LacZ-reporting viral particles pseudotyped with the HTLV envelope [23], we demonstrated that the envelope recognized and fused correctly with the membrane of endothelial cells. Expression of functional receptors at the cell surface are usually also demonstrated via the ability of target cells to form syncytia with infected lymphocytes [39]. Syncytia were indeed formed during co-cultures of infected lymphocytes and hCMEC/D3, and this could be prevented by addition of serum from an HAM/TSP patient, confirming the role of viral envelope in the process of cell-cell fusion. Similarly, the formation of syncytia could be prevented by addition of binding inhibitors directed to the previously described HTLV-1 receptors, demonstrating that it is a proper receptor-mediated entry. The death of cells forming syncytia was observed after one week in culture, as reported for other cell types [40], but p19 detection in the supernatant at later stages confirmed the persistent infection of endothelial cells. The production of viral proteins corresponds to that of the infectious virus, as shown using 293T-LTR-GFP reporter cells assays.

Of note, direct infection of cerebral endothelial cells may constitute a new mechanism of entry for the retrovirus into the CNS. In fact, endothelial cells and astrocytes are tightly associated at the BBB; infected endothelial cells could then transmit infectious particles to astrocytes, which have been shown to be susceptible to HTLV-1 infection both *in vitro* and *in vivo* [4],[26],[27],[41].

Finally, we analyzed the impact that infection of endothelial cells had on BBB integrity. At day 20 following co-culture with irradiated lymphocytes, the endothelial cells showed no cytopathic effect and no syncytia was observed. We demonstrated that HTLV-infected hCMEC/D3 cells could no longer form confluent monolayers resistant to molecular diffusion and cellular migration. Indeed, BBB dysfunction results in enhanced transendothelial migration of both HTLV-1-infected and uninfected lymphocytes. Both lymphocytes carrying HTLV-1 and tight junction opening could facilitate the spread of the virus and cytotoxic lymphocytes into the CNS. The mechanisms for such an alteration are not completely understood and may be multifactorial. We previously demonstrated that inflammatory cytokines produces by HTLV-1 infected lymphocytes induced BBB disruption, by increasing the expression and activity of the Myosin Light Chain Kinase (MLCK), which is transcriptionally regulated by the NF-κB pathway [8]. Since Tax expression induces cytokine secretion [42],[43],[44], disruption of the BBB could be a consequence of the autocrine or paracrine effects of proinflammatory cytokines secreted by HTLV-1 infected endothelial cells. This could explain why a low infection rate (<20%) of the cells is sufficient to significantly alter BBB-related functions. However, ML-7 treatment (an inhibitor of MLCK activity) of virally infected endothelial cells could not prevent such a disruption. This is consistent with the observations of McKenzie and Ridley who recently showed that MLCK activity is not required for long term modification of TJ protein expression, mediated by TNFα [45].

Further investigations could focus on possible interactions between viral Tax protein and proteins that constitute TJs. For example, the protein ZO-1 bears a PDZ domain [46] and Tax a PDZ binding domain [47] and suggest that these proteins interact with each other. This interaction could induce relocalization of ZO-1 and disorganize the TJ, or the protein could directly target the proteasome, as Tax does for the Retinoblastoma protein [48].

In conclusion, we have shown that endothelial cells, which constitute the BBB, are susceptible to infection by HTLV-1. This represents a new mechanism for BBB disruption in HAM/TSP, either directly as the expression of Tax induces a loss of BBB functions, or indirectly as the infected endothelial cells will be targeted by the immune system. Moreover, as the infection is productive, endothelial cells could allow entry of the virus into the CNS and facilitate the infection of astrocytes within the CNS parenchyma. Some may argue that the disruption of the BBB due to the infection of the endothelial cells seem to be a minor event in the natural course of HAM/TSP pathogenesis when compared to the proinflammatory cytokines [8]. However, considering this possibility should not be neglected for the design of potential new treatments. For example, the inhibition of the VEGF has been proposed previously as a way to prevent lymphocyte migration throughout the vasculature [49],[50]. Our results suggest that such a treatment should be envisioned with caution in the context of BBB, as it could increase the availability of the Neuropilin-1 to the virus and thereby, could facilitate infection of the BBB by HTLV-1.

## Materials and Methods

### Cells and tissues

The human Cerebral Microvascular Endothelial Cell line, hCMEC/D3, was immortalized after transduction with lentiviral vectors encoding the catalytic subunit of human telomerase hTERT and SV40 T antigen, as described previously [28]. hCMEC/D3 cells were grown in Endothelial Growth Medium-2 (EGM-2MV, Clonetics, Cambrex Biosciences, Workingham, UK) without hydrocortisone, on Biocoat tissue culture flasks (BD Biosciences, Bedford, MA).

MT-2 and C81-66 were used as HTLV-1-infected T cell lines. These are cell-lines derived from human umbilical cord blood T cells, following culture with irradiated cells from at ATLL patient. Both cell lines express the viral transactivator Tax-1, although C81-66 does not produce any viral particles. CEM and Jurkat were used as uninfected control T-cell lines. Non-adherent cell lines were grown in RPMI 1640 medium (Gibco BRL, Gaithersburg, MD) supplemented with 1 mM glutamine and 10% FCS.

The HEK epithelial cells containing an integrated HTLV-1 long terminal repeat (LTR) coupled to a green fluorescent protein (GFP) reporter gene (called herein 293T-LTR-GFP) [51] were grown in Dulbecco's modified Eagle's medium (Gibco BRL) supplemented with glutamine (1 mM), 100 U/ml penicillin, and 10% FCS.

Frozen tissue autopsy sections from a HAM/TSP patient were obtained as previously described [4]. For one experiment, we used paraffin embedded material from the thoracic spinal cord of a HAM/TSP patient from Chile, whose case has been previously described [52]. Frozen spinal cord tissues from uninfected control patients were obtained from the UK Multiple Sclerosis Tissue Bank and as previously described [53]. All tissue specimens were obtained in accordance with the respective hospital and national regulations and ethical rules.

### Immunohistochemistry, immunofluorescence and *in situ* hybridization

Fixed frozen (4% paraformaldehyde, PFA) and snap frozen blocks of tissues were sectioned serially at 10 µm using a cryostat; sections were then air-dried, and fixed in methanol before processing for immunohistochemistry. Sections were either labeled by immunoperoxidase technique (3-step procedure, DAKO, Glostrup, Denmark) or alcaline phosphatase technique (Vectastain, Vector Laboratories, CA, USA). Endogenous peroxidase was blocked with 2,5% hydrogen peroxidase in methanol, and non-specific labeling blocked with normal sera corresponding to the secondary antibody species. Sections were dehydrated in graded alcohols, cleared in xylene and coverslipped using permount. The primary antibodies used were a mouse antibody to Glut-1 (MAB 1418, R&D systems, Minneapolis, MN, USA), to NP-1 (clone A-12, Santa Cruz Biotechnology, Santa Cruz, CA, USA), or to Factor VIII (DAKO).

Cultures were fixed with 4% PFA. Staining with primary antibody was performed after incubation for 30 minutes with 10% normal goat serum and 0.05% saponin diluted in PBS. The following primary antibodies were used: mouse antibodies to HTLV-1-p24 (ab9081, Abcam, Cambridge, UK), to Glut-1 (R&D systems), to NP-1 (Santa Cruz Biotechnology). Specific secondary antibodies were coupled with Fluorescein (Vector Laboratories). After washes, preparations were mounted in DAPI-containing Vectashield medium (Vector laboratories).

ISH was performed on serial sections of frozen tissues by means of ^32^P antisense and sense riboprobes corresponding to the complete tax mRNA, as described elsewhere [54]. Sections were stained prior to ISH by immunoperoxidase using a rabbit polyclonal antibody against Glial Fribrillary Acidic Protein (anti-GFAP, DAKO).

### Flow cytometry analysis

Cells were analyzed for surface or intracellular receptor expression, with or without permeabilization with triton. Cells were resuspended in PBS-EDTA and incubated at 4°C for 30 min with a primary antibody to NP-1, Glut-1, HSPGs (clone F69-3G10, Seikagaku Corp., Tokyo, Japan) or HTLV-1 p24. Secondary antibody staining was performed by incubating the cells with fluorescein-isothiocyanate (FITC)-labeled antibody (Vector Laboratories) at 4°C for 30 min. Cells were washed twice with phosphate-buffered saline (PBS) and analyzed by using a FACScan flow cytometer and Cellquest software (Becton Dickinson, San Jose, CA, USA).

### Infection assays

Replication-defective LacZ retroviral vectors pseudotyped with either the HTLV (H-MLV), or amphotropic murine leukemia virus (A-MLV) envelope proteins were produced by transfection of 293T cells with Gag/Pol, Env, and LacZ plasmids as described elsewhere [23]. Target cells were plated on 24-well plates (5.10^4^) for 24 h and supernatants from H-MLV (1/5), or A-MLV (1/100) pseudotype-producing cells were added. Infectivity was assessed 48 h later by measuring the level of lacZ activity with the β-Gal reporter gene assay kit (Roche, France).

Syncytia were obtained after 24 h coculture of hCMEC/D3 cells and MT-2 infected lymphocytes (1∶1 ratio). The p24 staining was performed by an indirect immunofluorescence assay using the mouse anti-HTLV-1 p24 antibody on PFA fixed cells. To ascertain the endothelial origin of the syncytia, the hCMEC/D3 cells were prelabeled with CellTracker™ Red CMTPX (Molecular Probes, Invitrogen, Carlsbad, CA, USA). The preparations were visualized with a Zeiss Axiovert apparatus (Iena, Germany) or Leica DMRB (Wetzlar, Germany). The syncytia formation was inhibited by addition of dextran sulfate (100 µg/mL, Sigma Aldrich, St Louis, MO, USA), VEGF165 (50 ng/mL, R&D systems) or rabbit polyclonal antibody directed to Glut-1 (ab15309, Abcam; anti Cbl-b antibody H454 was used as an irrelevant antibody).

The extent of syncytia formation was assessed after 24 h in coculture by counting their numbers, as well as nuclei per syncytia, using Giemsa staining. All microscopic fields from 16 mm diameter coverslips were evaluated, from three different cultures.

For inhibition experiments, serum from a HAM/TSP patient or control serum (lacking HTLV-1 antibodies detected by Western blot assay) was added at the beginning of the coculture.

Chronically infected cells (MT-2 lymphocytes or C81-66 cells as control as they can not transmit infection) were irradiated at 10 Gy and washed twice with PBS to eliminate free radicals. hCMEC/D3 monolayers were cocultivated overnight at 37°C with the irradiated lymphocytes at a 1∶1 ratio and extensively washed three times with medium lacking serum. Human endothelial cell cultures were then maintained in normal culture medium. Aliquots of culture medium were collected at different time to detect viral proteins. HTLV-1 p19 was detected in culture media using the HTLV p19 antigen ELISA assay (Zeptometrix, Buffalo, NY, USA). In order to prevent the viral infection, 25 µM of AZT, an inhibitor of the reverse transcriptase, were added to the culture medium.

### Secondary infection

Secondary infection was performed as described previously [55]. Briefly, HCMEC/D3 cells were cocultured with irradiated MT-2 lymphocytes (or control C81-66 cells) for 15 days. The cells were then extensively washed and the medium replaced. Forty-eight hours later, the growth medium was collected and clarified by low-speed centrifugation (2,500 rpm for 5 min) then filtered through a 45-µm filter. The resulting media was then layered on a 20% glycerol gradient and the virus in it was pelleted by centrifugation in a SW28 rotor at 22,000 rpm for 2 h. The pellet was then resuspended in 200 µl of serum-free DMEM. 293T-LTR GFP indicator cells were incubated with 200 µl of the virus suspension in a total volume of 2 ml of serum-free DMEM for 2 h. Complete medium was then added and changed twice a week. One week later, the cells were fixed in 4% PFA, and visualized with a Zeiss Axiovert apparatus. The specificity of the signal was confirmed by addition of AZT into the culture medium.

### Immunoblot analyses

TJ proteins expression in HCMEC/D3 cells (cocultured for 15 days with MT-2 or C81-66 irradiated lymphocytes) was investigated by immunoblot analysis. Cells were washed twice with PBS, lysed in appropriate buffer (50 mM Tris-HCl,pH 7.4, 120 mM NaCl, 5 mM EDTA, 0.5% Nonidet P-40, 0.2 mM Na3VO4, 1 mM dithiothreitol, 1 mM phenylmethylsulfonyl fluoride) in the presence of a cocktail of protease inhibitors (Roche Applied Science, Indianapolis, IN, USA), and incubated on ice. Protein concentration was determined by the Bradford method (Bio-Rad, Hercules, CA, USA). Samples were loaded into 4–20% Tris/Gly gels (NOVEX, Invitrogen), subjected to SDS-PAGE, and transferred onto a nitrocellulose membrane (Immobilon-P, Millipore, Billerica, MA, USA). Following incubation with specific antibodies (rabbit anti ZO-1 and Occludin from Zymed or mouse anti-HTLV-p24 from AbCam) and extensive washing in PBS-Tween 0.05%, membranes were incubated with horseradish peroxidase-conjugated secondary antibodies (Vector Laboratories) and developed using either the SuperSignal WestPico or SuperSignal West Femto Chemiluminescent substrate kit (Pierce, Rockford, IL, USA). To ensure equal amount of protein loaded per well, membranes were stripped with the Re-blot Plus Kit (Chemicon International, Temecula, CA, USA) and reprobed with a specific anti β-tubulin antibody (Santa Cruz Biotechnology).

### Endothelial permeability assays

Permeability of hCMEC/D3 cell monolayers was measured using a method adapted from Dehouck et al. [56] and Romero et al. [30] on Transwell-ClearTM filters (polyester, 12 mm diameter, pore size 3 µm, Costar, Brumath, France). Briefly, 10^5^ cells/well were seeded on filters previously coated with rat-tail collagen I (BD Biosciences) and bovine plasma fibronectin (Sigma Aldrich). At confluence, hydrocortisone was added to EBM-2 medium as recommended by the manufacturer. After 24 hours, cells were used for experiments. Coculture experiments were set up by adding of 10^5^ lymphocytes to the endothelial monolayer in the presence or absence of inhibitors.

For the permeability test, the culture medium was replaced by DMEM without phenol red. FITC-labelled dextran (molecular weight 70 kDa, Sigma Aldrich) was added to the upper compartment and inserts were transferred sequentially at 5 minutes intervals from well to well for 30 min. The quantity of FITC-dextran that had diffused through the monolayer into the lower compartment at each time point was determined using a fluorescence multiwell plate reader (Wallac VictorTM 1420, PerkinElmer, Wellesley, MA, USA). The permeability coefficients of the endothelial monolayers were then calculated as previously described [30].

### T-lymphocyte migration through brain endothelial monolayers

Lymphocytes were labeled with CellTracker™ Green BODIPY® (Molecular Probes) according to manufacturer's instructions. Labeled lymphocytes were added to the upper chamber of Transwell-ClearTM insert filters (polyester, 12 mm diameter, pore size 3 µm, Costar) containing confluent hCMEC/D3 monolayers. After 24 hours at 37°C, the monolayer was extensively washed with PBS/EDTA, in order to collect lymphocytes adherent to each side of the membrane. Cells were lyzed using water. Fluorescence intensity was determined using a fluorescence multiwell plate reader.
